# Relatedness needs satisfaction or ego depletion? The effect of leisure nostalgia on life satisfaction

**DOI:** 10.3389/fpsyg.2025.1645603

**Published:** 2025-09-08

**Authors:** Dan Li, Juzhe Xi, Yeran Xu, Aifen Song, Yanwei Shi

**Affiliations:** ^1^College Student Mental Health Education and Consultation Center, Hainan Medical University, Haikou, China; ^2^Shanghai Key Laboratory of Mental Health and Psychological Crisis Intervention, Affiliated Mental Health Center (ECNU), School of Psychology and Cognitive Science, East China Normal University, Shanghai, China; ^3^Positive Education China Academy (PECA) of Han-Jing Institute for Studies in Classics, Juzhe Xi's Master Workroom of Shanghai School Mental Health Service, China Research Institute of Care and Education of Infants and Young Children, East China Normal University, Shanghai, China; ^4^Law School, East China Normal University, Shanghai, China; ^5^College Student Mental Health Education Center, Hainan Tropical Ocean University, Haikou, China; ^6^Department of Human Resource Management, Shanghai Normal University, Shanghai, China

**Keywords:** basic psychological needs theory, ego depletion, leisure nostalgia, life satisfaction, relatedness need satisfaction, self-regulatory capacity, self-regulation theory

## Abstract

**Background:**

Previous studies focused on the positive aspects of leisure nostalgia in relation to positive outcomes, overlooking its negative aspects and the possibility of negative effects. Based on the basic psychological needs theory and self-regulation theory, we tested whether relatedness need satisfaction and ego depletion mediated positive and negative effects of leisure nostalgia, respectively, and whether these mediation processes were moderated by self-regulatory capacity.

**Methods:**

We collected two waves of questionnaire data with a 1-month interval from 391 university students. The instruments used in this study included demographic characteristics and questionnaires of ego depletion, leisure nostalgia, life satisfaction, relatedness need satisfaction, self-regulatory capacity. Data analysis was performed using the SPSS and Mplus.

**Results:**

Leisure nostalgia was positively related to university student life satisfaction via higher relatedness need satisfaction (indirect effect = 0.11, 95% CI [0.052, 0.161]), and this indirect effect was stronger for students with higher vs. lower self-regulatory capacity (index = 0.03, 95% CI [0.003, 0.062]). By contrast, the mediating effect of ego depletion in the relationship between leisure nostalgia and lower life satisfaction was not significant (indirect effect = −0.01, 95% CI [−0.020, 0.011]).

**Conclusions:**

Findings from this study suggest that leisure nostalgia is a predominantly positive emotional experience that can promote university students' life satisfaction. The results have practical implications for designing programs to enhance university students' leisure nostalgia, self-regulation capacity, and life satisfaction.

## 1 Introduction

Lockdowns during the COVID-19 pandemic were associated with increases in social isolation, anxiety, stress, worry, and insomnia (Brooks et al., 2020; Rogowska et al., 2020; World Health Organization, 2022; Xin et al., 2020). Furthermore, the negative effects of pandemic-induced lockdowns on mental health persisted after restrictions were lifted (Pieh et al., 2021; Zhou et al., 2022). Similar to many other countries, universities in China implemented policies to enforce social distancing during the COVID-19 pandemic. Specifically, Chinese universities adopted stringent campus lockdowns, restricted students' movements, and limited social and leisure activities on campus to prevent virus transmission (Zhang et al., 2022a). These measures significantly disrupted students' daily lives and were associated with heightened feelings of loneliness (Zhou et al., 2022), as well as increased symptoms of anxiety and depression (Zhang et al., 2021; Zhou et al., 2021). For instance, a meta-analysis focusing on Chinese university students reported elevated levels of anxiety and depressive symptoms directly linked to prolonged campus restrictions (Zhang et al., 2021). Thus, pandemic-related policies within the Chinese higher education context had substantial and enduring impacts on students' mental health and overall wellbeing.

Under lockdown, many people felt nostalgic about former leisure activities (Cho, 2021; Gammon and Ramshaw, 2021). This leisure nostalgia involved poignant feelings of affection for their past leisure activities (Cho, 2020, 2021). Nostalgia for leisure activities was shown to be associated with higher wellbeing and life satisfaction during lockdown (Cho, 2020, 2023, 2021). In this respect, nostalgia about leisure activities could be similar to general nostalgia, which has been conceptualized as a psychological resource that enhances wellbeing (Onal et al., 2024; Sun et al., 2025a), including life satisfaction (Hepper and Dennis, 2022).

In the present study we addressed three gaps in this line of research. First, leisure nostalgia has been conceptualized in solely positive terms (Cho, 2020, 2023, 2021). This perspective is consistent with the basic psychological needs theory in that leisure nostalgia may satisfy basic psychological needs, and in turn promote life satisfaction (Deci and Ryan, 2000, 2002). However, like nostalgia in general (Huang and Uricher, 2024; Sedikides et al., 2008), leisure nostalgia is not always a purely positive emotional experience; it can also involve mixed feelings and ambivalence (Sun et al., 2025a). No studies to date have tested whether leisure nostalgia can have negative effects on life satisfaction.

Second, only several studies have investigated a possible mediator in the link between leisure nostalgia and life satisfaction. Specifically, (Cho 2024) tested found that individuals with high experience of leisure nostalgia reported higher levels of leisure satisfaction, which in turn predicted higher wellbeing. However, consistent with the self-regulation theory (Muraven and Baumeister, 2000), leisure nostalgia may deplete self-regulatory resources, leading to ego depletion which in turn reduces life satisfaction. In other words, leisure nostalgia may have the “double-edged sword” impact on individuals' life satisfaction. Based on basic psychological needs theory (Deci and Ryan, 2000, 2002) and self-regulation theory (Muraven and Baumeister, 2000), we tested the mediating roles of relatedness need satisfaction and ego depletion in the association between leisure nostalgia and life satisfaction, respectively.

Third, boundary conditions of the association between leisure nostalgia and life satisfaction have not been tested. We assert that self-regulation capacity is a moderator that affects the strength of this association. The self-regulation capacity is a skill that enables individuals to actively plan, control, evaluate, and adapt their thoughts, emotions, and actions in order to achieve their goals in an ever-changing environment (Zimmerman, 2000). Self-regulatory capacity has been shown to be a protective factor for university students coping with mental health challenges (Hofer et al., 2011), and it is possible that it was also a protective factor in the adverse conditions of lockdown during the COVID-19 pandemic. Self-regulation capacity should strengthen the first mediation process and weaken the second.

The present research will extend our understanding of the relationship between leisure nostalgia and life satisfaction by addressing these gaps in the literature. Specifically, we aimed to investigate whether leisure nostalgia can enhance university students' life satisfaction. In addition, we will examine the mechanisms through which leisure nostalgia may affect life satisfaction by testing the mediating roles of relatedness need satisfaction and ego depletion. Lastly, we will examine self-regulation capacity as a boundary condition of these processes.

Through these efforts, our study offers these contributions to the existing literature. Our study broadens the theoretical perspective in leisure nostalgia research by being the first to apply the basic psychological needs theory and self-regulation theory. This approach reveals that leisure nostalgia can produce both positive effects—enhancing life satisfaction through relatedness need satisfaction—and potential negative effects related to the ego depletion. In addition, the present study firstly explored self-regulatory capacity as a boundary condition, revealing that university students with higher self-regulatory capacity derive greater benefits from leisure nostalgia. These findings enhance our understanding of the conditions under which leisure nostalgia is most advantageous.

## 2 Hypothesis development

(Cho et al. 2019) introduced the concept of leisure nostalgia that a person might feel leisure nostalgia when they yearn for positive past leisure experiences. (Cho et al. 2019) proposed that leisure nostalgia consists of five dimensions. Specifically, leisure nostalgic experiences refer to a general personal leisure experience associated with leisure objects. Past memories related to favorite leisure objects can recall the nostalgic feeling. Leisure environment refers to the visible emotional connection to an experience, including the location, amenities, and equipment.

Leisure nostalgia in relation to socialization (leisure socialization) focuses on the nostalgic feelings that evoked by past experiences during interactions with others and the formation of new relationships. For example, friends participating in leisure activity with me evoke my leisure nostalgic feelings. Leisure nostalgia in relation to personal identity (leisure personal identity) is associated to an individual's sense of self and behavior within specific roles. For instance, attending a sporting event to support one's preferred team can reinforce one's role as a fan. The experience of being a dedicated supporter evokes a sense of leisure nostalgia. Leisure nostalgia concerning group identity (leisure group identity) is tied to an individual's affiliation with a specific group (Sun et al., 2025b). The distinctiveness and unique characteristics of the group can strengthen group identity, leading to nostalgic feelings (Cho et al., 2014). For example, individuals who have been members of a specific recreational group for an extended period of time (e.g., singing, hiking, and other groups) tend to develop a stronger attachment to positive memories of experiences with that group, which in turn increases their group identity and produces corresponding nostalgia.

Leisure nostalgia is a highly socialized emotional experience (Cho et al., 2019). When people experience leisure nostalgia, they often recall specific leisure events (such as listening to music, playing sports, or traveling) or being involved in these activities, all of which involve engagement with others. Leisure nostalgia may involve increased attention to social interactions, interpersonal relationships, feelings of love, and trust from others (Sedikides et al., 2016). It enables individuals to re-experience the positive interactions they have had with friends or family, thereby fostering feelings of care and support (Sun et al., 2025a). Consequently, it can be posited that leisure nostalgia may fulfill the need for relatedness, thereby enhancing university students' life satisfaction.

Basic psychological needs theory (BPNT; Ryan and Deci, 2017) provides a theoretical explanation for the mediating effect of interpersonal connection (relatedness need satisfaction) in the relationship between leisure nostalgia and life satisfaction. The theory proposes that humans actively seek out opportunities to fulfill key basic psychological needs, which are defined as innate organismic necessities and psychological nutriments—essential for growth, integrity, and wellbeing (Deci and Ryan, 2000, 2002). These needs are not merely cultural constructs but universal requirements; their satisfaction is critical for optimal psychological functioning, while their frustration is linked to diminished wellbeing (Ryan and Deci, 2017). Leisure nostalgia (Cho et al., 2019) emphasizes socialization and group identity: many leisure-nostalgic memories revolve around shared activities, social roles, and group affiliations. These elements map most directly onto the relatedness need.

Relatedness need is one of these core needs, referring to the ability to establish high-quality, reciprocal relationships in life, to feel cared for and respected by others, and to experience a sense of belonging within social groups (Deci and Ryan, 2002; Ryan and Deci, 2017). BPNT emphasizes that relatedness is not a superficial desire but a fundamental motivational force: individuals are inherently driven to connect with others in meaningful ways, as such connections foster a sense of security, validation, and purpose.

Leisure nostalgia, by its nature, is a deeply social emotional experience (Cho et al., 2019). It involves recalling past leisure activities—such as shared travel, group sports, or casual gatherings with friends—that are inherently intertwined with social interactions. These recollections reactivate memories of warmth, support, and mutual engagement, effectively simulating the experience of satisfying relatedness needs. In this way, leisure nostalgia acts as a “psychological resource” that revitalizes feelings of connection, even when current social contexts (e.g., pandemic-related restrictions) limit opportunities for in-person interaction (Puente-Díaz and Cavazos-Arroyo, 2021; Huang et al., 2024).

BPNT further posits that the satisfaction of basic psychological needs, including relatedness, directly contributes to enhanced life satisfaction by reinforcing a sense of coherence and fulfillment (Deci and Ryan, 2000). Consistent with the assumptions of the BPNT (Ryan and Deci, 2017), satisfaction of the relatedness need may be a resource that helps university students re-experience the feeling of being cared for and trusted by others (such as friends, classmates, family) during leisure activities. Basic psychological need satisfaction has been shown to be positively related to life satisfaction (Kara and Sarol, 2021; Leversen et al., 2012; Walker and Kono, 2018). Thus, there is evidence that leisure nostalgia is associated with relatedness need satisfaction, and that related need satisfaction is associated with life satisfaction. It appears possible that relatedness need satisfaction mediates the association between leisure nostalgia and life satisfaction.

While this mediation effect has not been examined to date, evidence from several studies has suggested that relatedness need satisfaction may mediate the link between leisure activities (rather than nostalgia for leisure activities) and life satisfaction. For example, (Kara and Sarol 2021) showed that psychological need satisfaction mediated the relationship between leisure activities and adults' life satisfaction. (Leversen et al. 2012) found related evidence that psychological need satisfaction due to leisure activities was associated with adolescents' life satisfaction. Building on the BPNT and related empirical findings, we formulated the following hypothesis:

**H1:** Relatedness need satisfaction will mediate the relationship between leisure nostalgia and life satisfaction.

Like general nostalgia (Newman and Sachs, 2020; Newman et al., 2020), leisure nostalgia might bring forth negative feelings such as loneliness and sadness. For example, if someone was nostalgic for enjoyable international travel, they may have experienced sadness due to pandemic-related travel restrictions. These negative emotions are not merely transient; they often involve a cognitive dissonance between the idealized past and the constrained present (Ohu et al., 2019), creating an ongoing psychological tension that demands regulatory effort. Significant effort is required to manage such negative emotions effectively—effort that draws on a finite pool of self-regulatory resources (Beal et al., 2005). Making this effort can deplete self-regulatory reserves (Beal et al., 2005), potentially resulting in adverse outcomes such as lower life satisfaction. In our study, we used ego depletion as an indicator of the depletion of self-regulatory resources.

Self-regulation theory provides a theoretical basis for exploring the mediating role of ego depletion in the relationship between leisure nostalgia and life satisfaction (Muraven and Baumeister, 2000). According to this theory, self-regulatory resources are not only limited but also domain-general: they support a range of behaviors requiring self-control, from emotion regulation to impulse inhibition and goal persistence (Baumeister et al., 2007). This resource pool operates like a “muscle”: while it can be temporarily depleted through exertion, it also has finite capacity in the short term (Muraven and Baumeister, 2000). When someone encounters stressful situations—including the emotional conflict inherent in leisure nostalgia—they recruit these resources to suppress negative affect, reframe distressing thoughts, or maintain emotional balance (Wenzel et al., 2024). Over time, repeated or intense use of these resources can lead to depletion (Koopmann et al., 2015), a state called ego depletion. When individuals experience ego depletion, they no longer have sufficient self-regulatory resources to engage in other behaviors requiring self-control, leaving them vulnerable to impulsive reactions, emotional ability, and reduced coping efficacy (Gissubel et al., 2018).

Applying self-regulation theory to our study, leisure nostalgia, when tinged with negative affect, functions as a chronic regulatory stressor. Unlike acute stressors that trigger a one-time resource expenditure, nostalgic longing for unattainable past leisure experiences (e.g., group sports, travel with friends) can become a recurring cognitive focus, especially during prolonged periods of social restriction. Each recurrence reignites the need to regulate disappointment or frustration, incrementally draining self-regulatory resources (Muraven and Baumeister, 2000). For university students, this process is compounded by the developmental context: emerging adulthood is marked by heightened sensitivity to social comparisons and unmet expectations, making the gap between nostalgic memories and present limitations particularly salient. Thus, leisure nostalgia may act as a persistent drain on self-regulatory resources, leading to ego depletion among university students.

Although there has been no empirical research on the relationship between leisure nostalgia and ego depletion, previous studies provided indirect evidence. For example, (Newman and Sachs 2020) found that general nostalgia was associated with more negative emotions and fatigue, which can both be manifestations of individual resource depletion. Therefore, based on self-regulation theory and relevant empirical research, we posit that leisure nostalgia may lead to ego depletion among university students.

Self-regulation theory further explains why ego depletion, in turn, reduces life satisfaction. The theory posits that depleted resources impair not only behavioral control but also subjective wellbeing: when self-regulatory capacity is low, individuals struggle to maintain positive self-evaluations, manage daily stressors, and pursue goals that contribute to life satisfaction (Baumeister et al., 2007). For university students, this means depleted resources may hinder their ability to stay motivated in academics, nurture social relationships, or engage in self-care—all of which are critical for perceived life quality. Additionally, ego depletion amplifies the salience of negative affect (Garrison et al., 2019): individuals with depleted resources are less able to downregulate feelings of sadness or frustration, leading to a more pessimistic appraisal of their lives (Boekhorst et al., 2017; Chen and Hsu, 2020). This pessimism, in turn, directly lowers life satisfaction.

Together, the assumptions of self-regulation theory, evidence of an association between leisure nostalgia and ego depletion, and evidence that ego depletion is related to life satisfaction, lead us to propose that leisure nostalgia may decrease university students' life satisfaction by increasing their ego depletion.

**H2:** Ego depletion will mediate the relationship between leisure nostalgia and low life satisfaction.

Self-regulation theory proposes that individual characteristics play a crucial role in mitigating the loss of self-regulatory resources, as some individuals have larger self-regulatory resource pools than others (Baumeister et al., 2007; Muraven and Baumeister, 2000). Researchers have proposed that a person's capacity for self-regulation as human quality because it enables us to survive and thrive (Greenhaus et al., 2012; Zimmerman, 2000).

Self-regulatory capacity is a skill that assists individuals in actively planning, controlling, assessing, and adjusting their thoughts, emotions, and behaviors to accomplish their objectives in an ever-evolving environment (Zimmerman, 2000). Specifically in this study, university students with high self-regulatory capacity were assumed to have more self-regulatory resources. Self-regulatory capacity has been shown to be a protective factor for university students in coping with mental health challenges (Hofer et al., 2011; Li et al., 2024). Those university students who have the capacity to regulate themselves through strategies that promote adaptive wellbeing and performance may be more likely to maintain mental health and happiness when facing adversity.

The enhancement perspective is the idea that a valuable resource can enhance the positive effects of another valuable resource (Greenhaus et al., 2012). For example, (Greenhaus et al. 2012) found that when employees perceived higher levels of organizational family support and partner support, the positive impact of family-supportive supervisor behaviors on employees' work-family balance was greater. We postulate that high self-regulatory capacity, as an important individual resource, can strengthen the positive effects of leisure nostalgia on relatedness need satisfaction and weaken the effects of leisure nostalgia on ego depletion.

Specifically, when university students have high self-regulatory capacity, engaging in leisure nostalgia can better satisfy their relational needs, thereby enhancing their life satisfaction. Additionally, high self-regulatory capacity helps replenish the self-regulatory resources depleted due to engaging in leisure nostalgia (i.e., ego depletion) for university students, reducing the impact of leisure nostalgia on ego depletion. In conclusion, we posit that self-regulatory capacity can moderate the relationship between leisure nostalgia, relational needs satisfaction, and ego depletion.

**H3a:** Self-regulation capacity will moderate the relationship between leisure nostalgia and relatedness need satisfaction, with the relationship being stronger when self-regulation capacity is high.

**H3b:** Self-regulation capacity will moderate the relationship between leisure nostalgia and ego depletion, with the relationship being weaker when self-regulation capacity is high.

The above hypotheses constitute two moderated mediation models (Hayes, 2013) in which the mediating effects of relatedness need satisfaction and ego depletion in the relationship between leisure nostalgia and life satisfaction are moderated by self-regulation capacity. Specifically, university students with high self-regulation capacity are able to obtain more resources from leisure nostalgia and to more effectively use those resources to satisfy relatedness need, thereby enhancing high life satisfaction. In addition, individuals with high self-regulation capacity have more resources to replenish those resources depleted by engaging in leisure nostalgia, thereby alleviating the negative impact of leisure nostalgia on ego depletion and ultimately enhancing life satisfaction. Based on the conceptual arguments, we proposed the following moderated mediation hypothesis:

**H4a:** Self-regulation capacity will moderate the indirect effect of leisure nostalgia on life satisfaction through relatedness need satisfaction, with the indirect effect being stronger when self-regulation capacity is high.

**H4b:** Self-regulation capacity will moderate the indirect effect of leisure nostalgia on life satisfaction through ego depletion, with the indirect effect being weaker when self-regulation capacity is high.

**Figure 1:**
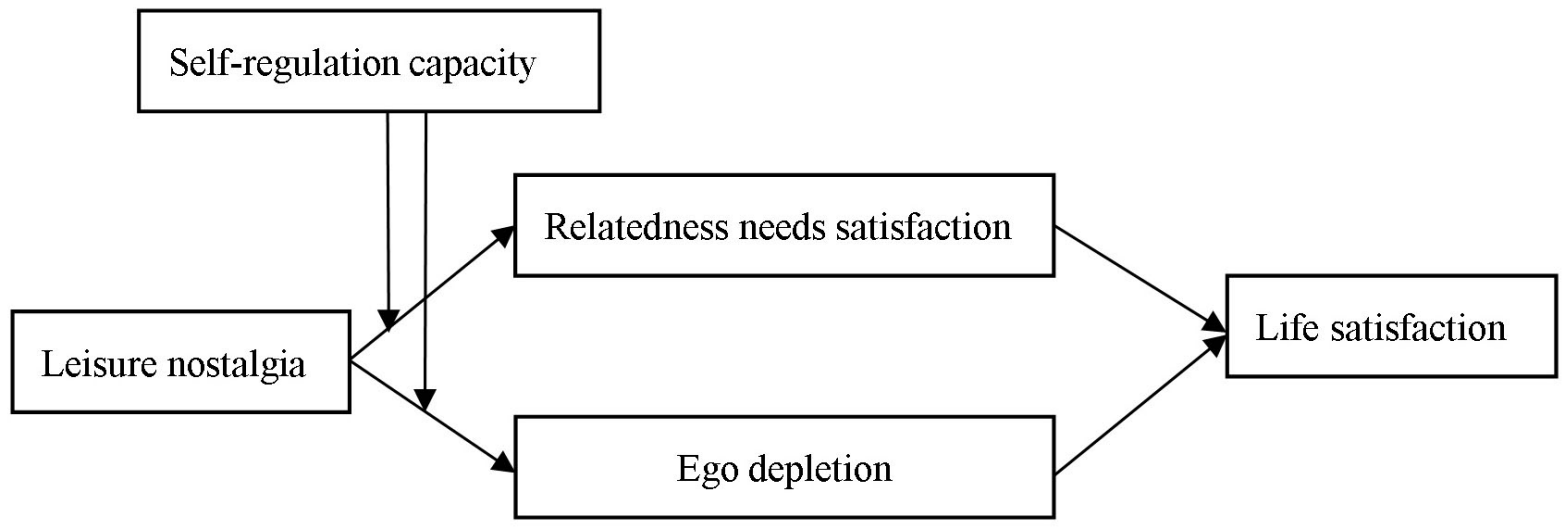
Hypothesized moderate mediation model.

Figure 1 provides a summary of the proposed relationships in the present study.

## 3 Materials and methods

### 3.1 Participants and procedure

We recruited 391 university students from three universities in the south of China using convenience sampling. The research opportunity was made known to students by instructors at the aforementioned universities. In addition, we asked the students in these classes to recommend other students. Specifically, the participant told the other students about the study, and the other students then contacted the researcher. At the end of the recruitment period, the research assistants added all participants to a WeChat group; the informed consent form and questionnaires were then distributed through links. Participants were informed that participation was voluntary, their responses were confidential, and they could withdraw at any time without penalty.

To mitigate common method bias, the participants completed some questionnaires at the beginning of the study and completed other questionnaires 1 month later. Based on previous research and considering the relatively short duration of leisure nostalgia effects, at the first time point we collected demographic information and responses to questionnaires that assessed leisure nostalgia, self-regulation capacity, and control variables. At the second time point we collected responses to measures of relatedness need satisfaction, ego depletion, and life satisfaction. Participants received research credits and a 10 RMB WeChat red packet (gift card) after completing both surveys. The two sets of questionnaires were matched using student ID numbers provided by the participants.

In the first round of data collection, we distributed a total of 600 questionnaires and received 528 valid responses (88%). In the second round of data collection we only distributed questionnaires to participants who had completed the first survey, and collected 420 valid questionnaires. We included lie a detection question in the questionnaire (Please select A) and found 29 incorrect responses (they select B), who were excluded. In the end, we obtained 391 valid questionnaires (74.05%). Among these, there were 144 men and 247 women; 262 freshmen, 62 sophomores, 65 juniors, and 2 seniors. The participants' ages ranged from 17 to 22, with an average age of 18.88 ± 0.72.

### 3.2 Measures

#### 3.2.1 Leisure nostalgia

The 33-item Leisure Nostalgia Scale (Cho et al., 2019) is a Chinese-language measure of leisure nostalgia. The questionnaire includes five subscales: leisure experience (five items; e.g., “My exciting leisure experience in the past evokes my nostalgic feelings”), environment (seven items; e.g., “The leisure activity equipment I used evokes my nostalgic feelings”), socialization (six items; e.g., “Positive memories shared with others during my favorite leisure activity evoke my nostalgic feelings”), personality identity (seven items; e.g., “Positive feelings about myself as a lover of my favorite leisure activity evoke my nostalgic feelings”), and group identity (seven items; e.g., “Shared memories which affected my group identity at the leisure place evoke my nostalgic feelings”). The participants were asked to express their level of agreement with each statement using a Five-point Likert scale (1 = strongly disagree, 5 = strongly agree), where higher average scores reflect greater leisure nostalgia.

The Chinese version of the 33-item Leisure Nostalgia Scale (LNS) was rigorously validated in a pre-study conducted as part of the first author's doctoral dissertation project underpinning this research. In that pre-study, data were collected from a large sample of 1,835 Chinese university students and conducted rigorous psychometric testing: the scale demonstrated excellent internal consistency, with Cronbach's α ranging from 0.93 to 0.96 across its five subscales (leisure experience, environment, socialization, personal identity, and group identity), confirming high reliability. Confirmatory factor analysis (CFA) using Mplus 8.0 supported the five-factor structure of the Chinese version, with fit indices: χ^2^/df = 10.47, CFI = 0.93, TLI = 0.92, RMSEA = 0.07. In the present study, the Cronbach's α coefficient for the leisure nostalgia scale was 0.96. Confirmatory factor analysis was conducted to assess the construct validity of the scale. The results indicated a good model fit, with χ^2^/df = 1.82, RMSEA = 0.05, TLI = 0.92, and CFI = 0.93, all of which meet commonly accepted criteria.

#### 3.2.2 Relatedness need satisfaction

We used a subscale of the Basic Psychological Needs Scale (Gagné, 2003) to measure relatedness need satisfaction. There are eight items, such as “People around me care about me.” Participants used a Five-point Likert scale (1 = strongly disagree, 5 = strongly agree) to rate each item The average score was calculated after reverse-coding three items, with higher scores indicating higher relatedness need satisfaction. In our study, the Cronbach's α coefficient for the relatedness need satisfaction scale was 0.77, aligning with prior validation studies in Chinese samples (e.g., Yang et al., 2021, who reported α = 0.80). Confirmatory factor analysis was conducted to examine the construct validity and the results demonstrated acceptable fit (χ^2^/df = 4.49, RMSEA = 0.09, TLI = 0.90, CFI = 0.94).

#### 3.2.3 Ego depletion

We used the five-item Ego Depletion Scale (Bertrams et al., 2010) to assess ego depletion. A sample item is “I feel like my willpower is gone.” Each item is rated on a Four-point Likert scale (1 = not at all, 4 = a great deal), with higher scores indicating higher ego depletion. In our study, the Cronbach's alpha of the scale was 0.91, aligning with prior validation studies in Chinese samples (e.g., Zhang et al., 2022b, who reported α = 0.90). Confirmatory factor analysis was conducted to examine the construct validity and the results demonstrated acceptable fit (χ^2^/df = 3.16, RMSEA = 0.07, TLI = 0.99, CFI = 0.98).

#### 3.2.4 Self-regulation capacity

The Self-Regulation Capacity Scale (Schwarzer et al., 1999) was used to measure self-regulation capacity. There are 10 items, each rated on a Five-point Likert scale (1 = strongly disagree, 5 = strongly agree). Example items are “I can control my thoughts so that they do not interfere with my current study tasks” and “A number of thoughts and emotions make it difficult for me to concentrate on my studies” (reverse scored). After reverse scoring three items, the final score was the average of all item scores, with higher scores indicating higher self-regulation capacity. This scale has shown good reliability and validity in a Chinese sample (Shi et al., 2023). In our study, the Cronbach's alpha of the scale was 0.76. Confirmatory factor analysis was conducted to examine the construct validity and the results demonstrated acceptable fit (χ^2^/df = 3.26, RMSEA = 0.07, TLI = 0.86, CFI = 0.90).

#### 3.2.5 Life satisfaction

We used the five-item Life Satisfaction Scale (Diener et al., 1985) to assess life satisfaction. An example item is “In most ways, my life is close to my ideal.” Each item is rated on a Seven-point Likert scale (1 = strongly disagree; 7 = strongly agree). In our study, the Cronbach's alpha of the scale was 0.87, aligning with prior validation studies in Chinese samples (e.g., Shi et al., 2020, who reported α = 0.86). Confirmatory factor analysis was conducted to examine the construct validity and the results demonstrated acceptable fit (χ^2^/df = 1.82, RMSEA = 0.05, TLI = 0.99, CFI = 0.99).

#### 3.2.6 Control variables

Research on nostalgia suggests that an individual's gender and age influence the effects of nostalgia (Juhl et al., 2021; Sedikides et al., 2016). Therefore, in this study, the gender and age of the students were included as control variables in the statistical analyses.

### 3.3 Analysis strategies

Prior to the main analyses, the dataset underwent thorough screening to ensure data quality and integrity. Invalid responses were identified and removed based on a lie detection item embedded in the questionnaire (i.e., a question instructing respondents to select a specific answer), leading to the exclusion of 29 participants who failed this check. Subsequently, descriptive analyses and standardized scores were examined to identify potential outliers. No extreme values were found that met criteria for exclusion.

First, confirmatory factor analysis was performed using Mplus 8.3 (Muthén and Muthén, 2010) to evaluate the discriminant validity of all variables. Subsequently, path analysis was conducted to test our hypotheses. Leisure nostalgia was specified as the independent variable, self-regulation capacity as the moderator, ego depletion and relatedness need satisfaction as mediators, and life satisfaction as the outcome variable. Control variables (age and gender) were included as predictors of the mediators and the dependent variable.

Before analysis, both the independent variable and moderator were grand-mean centered. Interaction effects were visualized, and simple slopes were examined for different levels of self-regulation capacity (+1 SD above and below the mean). We estimated 95% CIs using estimates from 5,000 bootstrapped samples for the hypothesized relationships to determine their significance.

### 3.4 Results

#### 3.4.1 Common method variance

Harman's single-factor test was conducted to detect CMV. The first factor accounted for 25.93% of the variance among variables, which is less than the 50% (Podsakoff et al., 2012). Thus, this result suggested that common method variance was minimal.

#### 3.4.2 Descriptive statistics

To address potential concerns regarding attrition bias, this study conducted a thorough attrition analysis. Following the recommendations of (Carlson et al. 2019), we examined whether significant differences existed in demographic characteristics and independent variables between participants who completed only the Time 1 questionnaire (*n* = 137) and those who participated at both Time 1 and Time 2 (*n* = 391). Independent-samples *t*-tests indicated no significant differences between the two subgroups in leisure nostalgia [*t*_(526)_ = 1.01, *p* = 0.31] or age [*t*_(526)_ = 1.01, *p* = 0.32]. A chi-square test further revealed no significant difference in gender distribution across the subgroups (χ^2^ = 1.67, *p* = 0.22).

Additionally, following the guidance of (Goodman and Blum 1996) and previous research (Belschak et al., 2020), we conducted a multiple logistic regression to further assess potential non-random attrition. Participants were classified as stayers (those who responded at both Time 1 and Time 2) or leavers (those who responded only at Time 1). The independent variables were the key variables measured at Time 1. The regression results showed no evidence of systematic attrition effects for leisure nostalgia (*B* = −0.09, *SE* = 0.09, *p* = 0.31), gender (*B* = 0.05, *SE* = 0.28, *p* = 0.86), or age (*B* = −0.08, SE = 0.08, *p* = 0.32). These findings suggest that attrition occurred at random with respect to the main variables in our model. Accordingly, the matched dataset of 391 university students who completed both Time 1 and Time 2 surveys was used for hypothesis testing.

Table 1 presents the means, standard deviations, and intercorrelations for all variables. As expected, leisure nostalgia was positively related to relatedness need satisfaction (*r* = 0.23, *p* < 0.001) and life satisfaction (*r* = 0.25, *p* < 0.001); relatedness need satisfaction was positively related to life satisfaction (*r* = 0.45, *p* < 0.001); and ego depletion was negatively related to life satisfaction (*r* = −0.13, *p* < 0.01). Contrary to expectations, the relationship between leisure nostalgia and ego depletion was not significant (*r* = −0.04, *p* > 0.05).

**Table 1:** Means, standard deviations, and correlations among study variables.

**Variables**	***M***	***SD***	**1**	**2**	**3**	**4**	**5**	**6**	**7**
1. Gender	–	–	–						
2. Age	18.22	0.72	−0.14	–					
3.Self-regulation capacity	3.26	0.49	−0.06	0.04	–				
4. Leisure nostalgia	5.26	0.91	0.07	0.06	0.16^**^	–			
5.Relatedness need satisfaction	3.51	0.58	0.01	0.03	0.42^***^	0.23^***^	–		
6. Ego depletion	2.80	0.93	−0.04	0.07	−0.41^***^	−0.04	−0.51^***^	–	
7. Life satisfaction	4.51	1.51	−0.06	0.07	0.29^***^	0.25^***^	0.45^***^	−0.13^**^	–

^*^p < 0.05; ^**^p < 0.01; ^***^p < 0.001.

#### 3.4.3 Hypothesis testing

**Figure 2:**
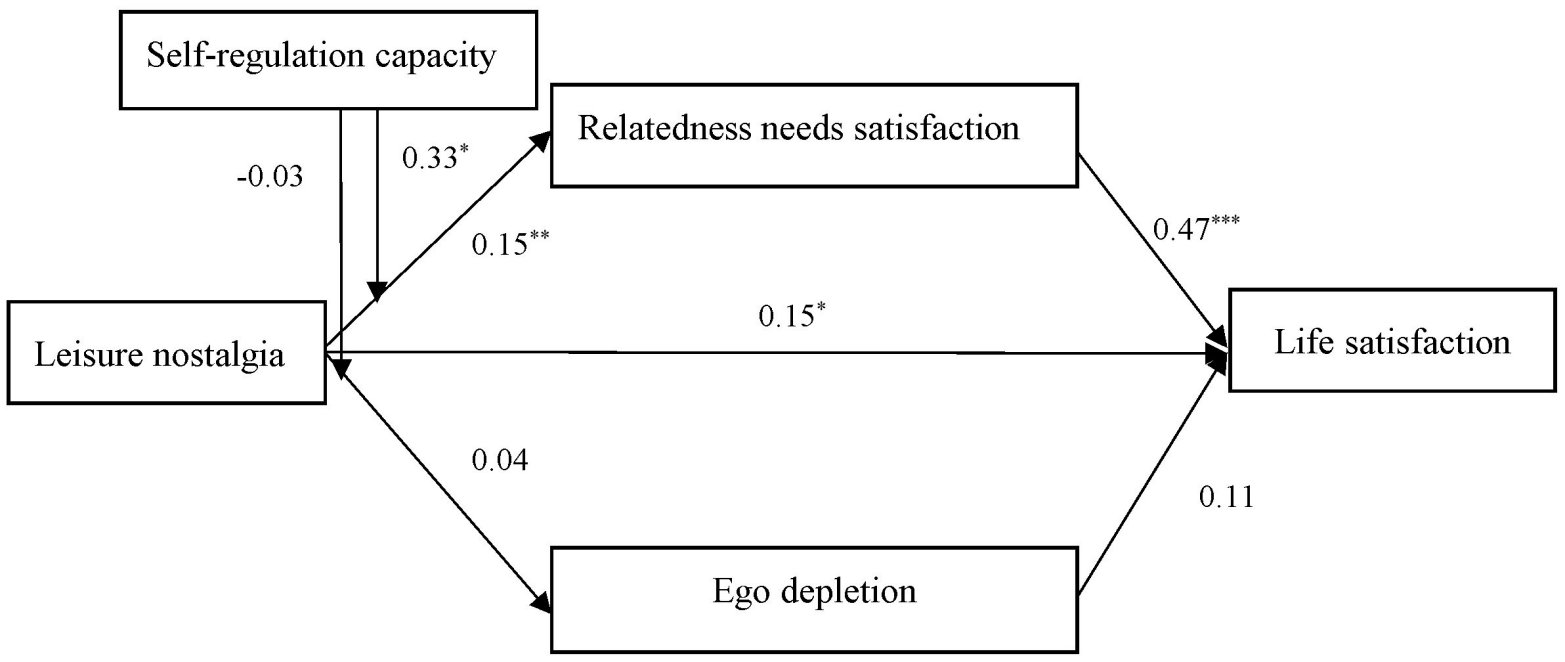
The moderate mediation model. All the reported parameters are standardized; *p < 0.05; **p < 0.01; **p < 0.001.

Table 2 and Figure 2 showed the results of hypothesis. Hypothesis 1 was that relatedness need satisfaction will mediate the relationship between leisure nostalgia and greater life satisfaction. Leisure nostalgia positively predicted relatedness need satisfaction (Equation 1, β = 0.23, *SE* = 0.05, *p* < 0.001), and relatedness need satisfaction positively predicted life satisfaction (Equation 5, β = 0.47, *SE* = 0.06, *p* < 0.001). Leisure nostalgia had a significant indirect effect on life satisfaction via relatedness need satisfaction (indirect effect = 0.11, 95% CI [0.052, 0.161]). Thus, Hypothesis 1 was supported.

**Table 2:** Regression results for meditation effect of relatedness need satisfaction and ego depletion and moderated meditation effect.

**Variables**	**Relatedness need satisfaction (T2)**				**Ego depletion (T2)**				**Life satisfaction (T2)**			
	**Equation 1**		**Equation 2**		**Equation 3**		**Equation 4**		**Equation 5**		**Equation 6**	
	β	***SE***	β	***SE***	β	***SE***	β	***SE***	β	***SE***	β	***SE***
Gender	−0.01	0.10	0.03	0.05	−0.06	0.11	−0.06	0.05	−0.14	0.09	−0.07	0.04
Age	0.02	0.07	−0.01	0.05	0.09	0.07	0.08	0.05	0.05	0.07	0.04	0.05
Leisure nostalgia	0.23^***^	0.05	0.15^**^	0.05	−0.04	0.06	0.04	0.05	0.15^**^	0.05	0.15^**^	0.05
Relatedness need satisfaction									0.47^***^	0.06	0.47^***^	0.05
Ego depletion									0.11	0.07	0.11	0.06
Self-regulation capacity			0.41^***^	0.04			−0.43^***^	0.05				
Leisure nostalgia × self-regulation capacity			0.10^*^	0.05			−0.04	0.05				

^*^p < 0.05; ^**^p < 0.01; ^***^p < 0.001.

Hypothesis 2 was that ego depletion will mediate the relationship between leisure nostalgia and lower life satisfaction. Leisure nostalgia did not significantly predict ego depletion (Equation 3, β = −0.04, *SE* = 0.06, *p* > 0.05), and ego depletion did not significantly predicted life satisfaction (Equation 5, β = 0.11, *SE* = 0.07, *p* > 0.05). The indirect effect of leisure nostalgia on life satisfaction via ego depletion was not significant (indirect effect = −0.01, 95% CI [−0.020, 0.011]). Thus, Hypotheses 2 was not supported.

**Figure 3:**
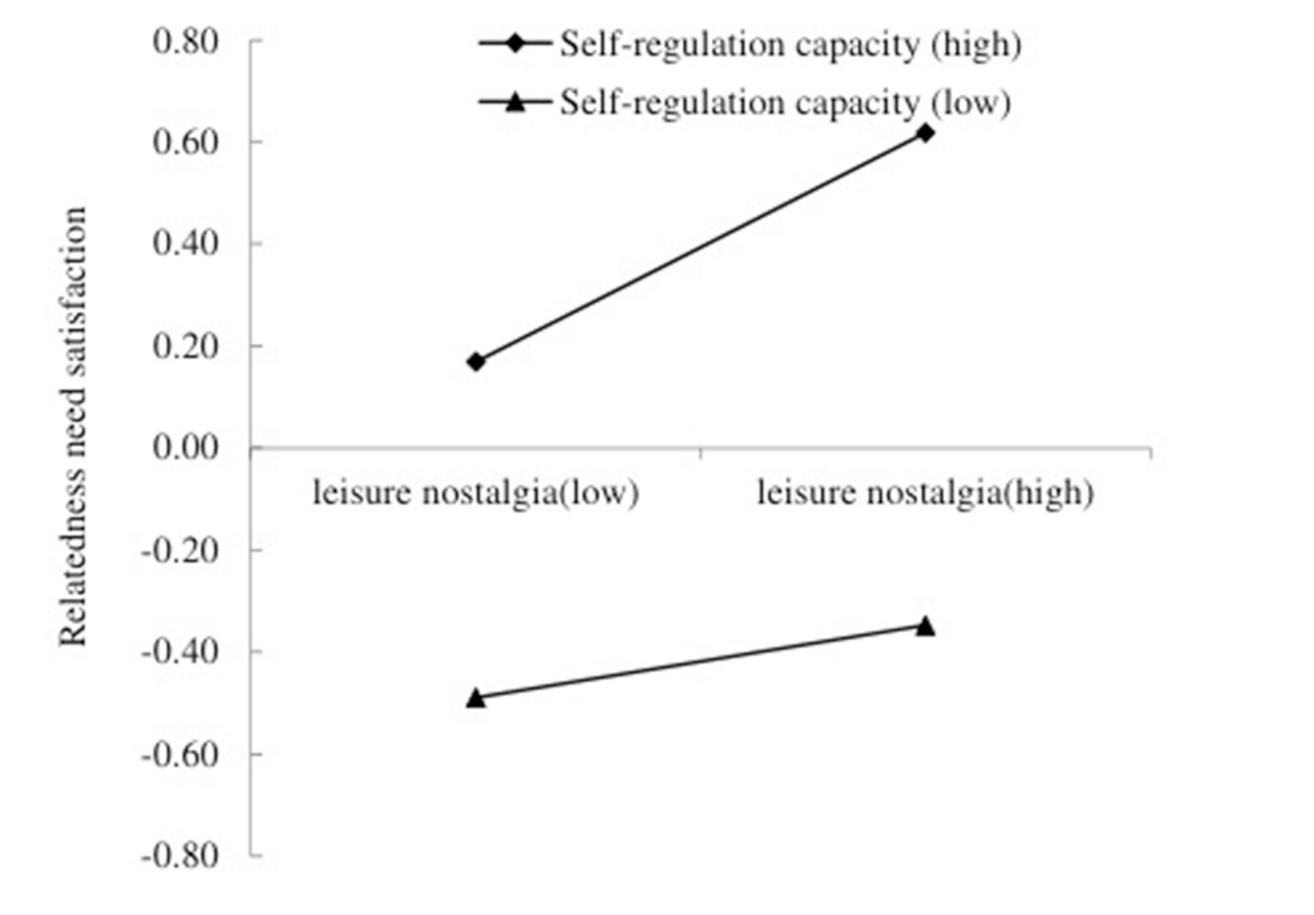
Self-regulation capacity moderates the relation between leisure nostalgia and relatedness need satisfaction.

We then tested the two moderated mediation models. Hypothesis 3a was that self-regulation capacity will moderate the association between leisure nostalgia and relatedness need satisfaction. The interaction between leisure nostalgia and self-regulation capacity was a positive and significant predictor of relatedness need satisfaction (Equation 2, β = 0.10, *p* < 0.05, 95% CI [0.003, 0.143]). The association between leisure nostalgia and relatedness need satisfaction was positive and significant when self-regulation capacity was high (β = 0.22, *p* < 0.001, 95% CI [0.109, 0.339]), but non-significant when self-regulation capacity was low (β = 0.07, *p* > 0.05, 95% CI [−0.044, 0.187]). Thus, Hypothesis 3a was supported. See Figure 3.

Hypothesis 3b was that self-regulation capacity will moderate the association between leisure nostalgia and ego depletion. The interaction of leisure nostalgia and self-regulation capacity in predicting ego depletion was not significant (Equation 4, β = −0.04, *p* > 0.05). Thus, Hypotheses 3b were not supported.

In addition, the results showed that the indirect effect of leisure nostalgia on life satisfaction via relatedness need satisfaction will be positively and significantly moderated by self-regulation capacity (index = 0.03, SE = 0.02, 95% CI [0.003, 0.062]). We also estimated the indirect effect of leisure nostalgia on life satisfaction through relatedness need satisfaction at higher (+1 *SD*) and lower levels (−1 *SD*) of self-regulation capacity. Specifically, the indirect effect was significant when self-regulation capacity was high (conditional indirect effect = 0.09, 95% CI = [0.046, 0.146]), but was not significant when self-regulation capacity was low (conditional indirect effect = 0.02, 95% CI = [−0.189, 0.084]). Thus, Hypothesis 4a was supported.

Hypothesis 4b was that the indirect effect of leisure nostalgia on life satisfaction via ego depletion will be significantly moderated by self-regulation capacity, but the moderated mediation model was not significant (index = 0.01, SE = 0.01, 95% CI [−0.004, 0.022]). Thus, Hypotheses 4b were not supported.

## 4 Discussion

Drawing on the basic psychological needs theory (Deci and Ryan, 2000, 2002) and self-regulation theory (Muraven and Baumeister, 2000), leisure nostalgia might have positive and negative effects on life satisfaction. The results showed that relatedness need satisfaction mediated the relationship between leisure nostalgia and higher life satisfaction, and self-regulation capacity moderated this mediation process. Specifically, the indirect effect was stronger for individuals with high self-regulation capacity than those with low self-regulation capacity. However, the mediating role of ego depletion in the relationship between leisure nostalgia and life satisfaction was not significant. Together, the results suggest that leisure nostalgia is important in enhancing life satisfaction.

Although data were collected during the COVID-19 pandemic and pandemic-related social restrictions may have increased leisure nostalgia, the core psychological mechanism—leisure nostalgia satisfying basic psychological needs for continuity and relatedness—likely generalizes beyond this context. Leisure nostalgia remains important for maintaining a sense of belonging and psychological continuity in non-pandemic settings, underscoring its enduring relevance for human wellbeing.

### 4.1 Theoretical contributions

Several contributions to the leisure nostalgia literature are made in the current study. First, whereas previous research (Cho et al., 2019; Cho, 2021) conceptualized the effects of leisure nostalgia only in positive terms, leisure nostalgia is a paradoxical emotional experience with mixed feelings of joy and sadness, and may have both positive and negative effects. Although we tested both ideas, our results supported a positive model of leisure nostalgia rather than a negative model. The results did not support the self-regulation theory; factors such as self-regulation and ego depletion may not be relevant in the association between leisure nostalgia and life satisfaction. These findings showed that leisure nostalgia, as an experience dominated by positive emotions, positively influences college students' life satisfaction. This aligns with prior research demonstrating leisure nostalgia's benefits for life satisfaction, leisure satisfaction, and work performance (e.g., Cho et al., 2019, 2020). It also supports broader findings that nostalgia generally promotes positive emotions and enhances both work and life outcomes (e.g., Newman and Sachs, 2020; Zhou et al., 2022).

Second, we examined a mechanism of the effects of leisure nostalgia on life satisfaction based on basic psychological needs theory and self-regulation theory. Specifically, we found that leisure nostalgia enhanced university students' life satisfaction by satisfying their need for relatedness. This finding is consistent with recent work by (Sun et al. 2025a). (Sun et al. 2025a) reported that Western exchange students in South Korea sought nostalgic leisure experiences that evoked warmth and familiarity, fostering belonging. Similarly, (Sun et al. 2025b) found that Chinese table tennis players used nostalgic activities to integrate into local groups, expressing a desire to connect through shared memories. Both studies highlight leisure nostalgia as a social resource that enhances emotional connection and belonging, which aligns with our result that it boosts life satisfaction by fulfilling relatedness needs.

Contrary to expectations, ego depletion did not mediate the relationship between leisure nostalgia and life satisfaction. According to the self-regulation theory (Muraven and Baumeister, 2000), individuals engage in self-regulation during the process of leisure nostalgia. Self-regulation requires resource consumption, and resources could become depleted over time. We assert that resources are not always depleted, because self-regulation can produce benefits that counteract the consumption of resources. Self-regulation allows a person to pursue goals and to experience positive emotions, a sense of achievement, and self-efficacy. In other words, self-regulation can sometimes be an active “investment of resources” that reduces the likelihood of ego depletion.

Chickering's theory also offers a developmental lens to explain why ego depletion did not mediate the relationship between leisure nostalgia and life satisfaction: Chickering's seven vectors highlight that college students, in a stage of actively developing competence and managing emotions (Chickering and Reisser, 1993), may see self-regulation during leisure nostalgia as a deliberate effort to practice emotional management or strengthen social bonds—aligning with their growth tasks and generating positive feedback that offsets resource expenditure.

Furthermore, methodological issues may hinder detection. The 1-month two-wave design missed ego depletion's transience (peaking then fading; Gissubel et al., 2018), masking mediation. Future research could use experience sampling or daily diaries to capture short-term ego depletion dynamics, assessing variables multiple times daily. This aligns with self-regulation theory (Muraven and Baumeister, 2000) and may reveal undetectable within-person effects.

In addition, the current study offers a new theoretical perspective to the field of leisure nostalgia research by incorporating self-regulation theory and basic psychological needs theory. When investigating the impact of leisure nostalgia, researchers predominantly relied on theoretical frameworks such as the theory of emotional appraisal (Lazarus, 2001), the goal-directed behavior model (Perugini and Bagozzi, 2001), and the broaden-and-build theory of positive emotions (Fredrickson, 2001). Our study incorporates basic psychological needs theory and self-regulation theory into the research on leisure nostalgia to reveal both the positive and negative effects of leisure nostalgia on university students' life satisfaction. This enriches research on basic psychological needs satisfaction theory and self-regulation depletion theory.

Third, our results are consistent with the enhancement perspective that one beneficial resource can amplify the positive impacts of another valuable resource (Greenhaus et al., 2012). As expected, the indirect effect of leisure nostalgia on life satisfaction through relatedness need satisfaction was stronger for university students with high vs. low self-regulation capacity. University students with high self-regulation capacity presumably have more resources and are more likely to benefit from leisure nostalgia. In this group, leisure nostalgia may be especially helpful as a way to satisfy their need for relatedness, and ultimately increase life satisfaction. To our knowledge, this is the first study to examine boundary conditions of the relationship between leisure nostalgia and life satisfaction, and the results extend our understanding of when leisure nostalgia is most beneficial.

### 4.2 Practical implications

The results of the current study have several practical implications for improving university students' life satisfaction. First, leisure nostalgia was positively related to university students' life satisfaction, suggesting that helping university students to experience leisure nostalgia could be beneficial. One intervention designed to promote general nostalgia helped enhance life satisfaction during and after the period of epidemic lockdown (Dennis and Ogden, 2022). Specific interventions involve defining nostalgia and prompting participants to recall a nostalgic event from their past before the lockdown. Specifically, they are asked to reflect on a particular event that evokes the strongest feelings of nostalgia. Based on the results of this general nostalgia intervention and the results of our study, we suggest that universities implement training programs for students to enhance their life satisfaction by engaging in leisure nostalgia. For example, university mental health centers could host monthly 60–90 min “leisure nostalgia workshops,” where students complete guided reflection worksheets and share memories in small groups to rebuild social connections.

Our results also suggest the possibility that university students' interactions with school personnel can promote students' life satisfaction by satisfying the need for relatedness. Previous research has shown that mindfulness-based intervention can satisfy relatedness need (Malboeuf-Hurtubise et al., 2018), and a similar program could be implemented with university students. In addition, university students with low life satisfaction can be encouraged to actively seek social support from friends and family to satisfy their relatedness needs.

Finally, the results suggest that enhancing university students' self-regulation capacity may predict higher life satisfaction. Students with higher self-regulation capacity may be able to increase their life satisfaction by using leisure nostalgia to satisfy relatedness needs. In addition, previous research showed that self-regulation interventions can improve individuals' self-regulation capacity (Dubuc-Charbonneau and Durand-Bush, 2015). The self-regulation interventions described by (Dubuc-Charbonneau and Durand-Bush 2015) follow a structured process: starting with baseline assessments and collaborative goal-setting (e.g., using nostalgic leisure memories to foster social connections); then providing skill training in cognitive restructuring, emotion regulation (such as mindfulness and journaling), and behavioral monitoring; next, encouraging daily practice with ongoing feedback to reinforce effective strategies and address challenges; and finally, gradually tapering the intervention to promote independent skill use across contexts, with follow-up assessments to ensure lasting improvements in self-regulation and related outcomes like life satisfaction. Based on this, school personnel could provide self-regulation interventions to university students to enhance their self-regulation capacity, which will provide self-regulation resources to cope with life and academic problems and experience higher levels of life satisfaction.

### 4.3 Limitations and future research

Several limitations of the current study should be considered in future research. First, data were collected at separate time points to mitigate mono-method bias, but the exclusive use of self-report measures raises similar concerns. Nevertheless, the results indicated no significant methodological bias. Furthermore, many of the variables, such as leisure nostalgia, ego depletion, relatedness need satisfaction, and life satisfaction are subjective experiences and therefore self-report is an appropriate approach to assessment, but future research could incorporate diverse data sources to deepen our understanding of life satisfaction. For example, integrating life satisfaction ratings from both university students and close friends could provide valuable insights.

A further limitation is the two-wave design, which limits robust longitudinal inferences. The 1-month interval and limited measurement points restrict our ability to fully understand the stability and dynamics of the proposed relationships over time. For example, we cannot definitively determine whether the mediating role of relatedness need satisfaction endures beyond 1 month, nor can we rule out reverse causality (e.g., life satisfaction influencing subsequent leisure nostalgia) over longer periods, thus weakening causal inferences regarding directionality. Future studies should employ multi-wave designs (e.g., three or more waves over 3–6 months) to test the stability of the mediating effect, explore reciprocal relationships using cross-lagged models, and clarify whether leisure nostalgia's impact on life satisfaction is temporary or lasting.

Second, a notable limitation is the demographic imbalance in our sample, including uneven class year distribution and a majority of female participants (63.2%). Given college students' rapid developmental changes (e.g., in self-regulation, social integration) and documented gender differences in leisure preferences (Henderson et al., 2002), findings may lack generalizability, though gender minimally influenced core relationships. Future research could test whether the effect of vary by class year, developmental stage, or gender to enhance robustness.

Third, we tested the mediating roles of relatedness need satisfaction and ego depletion, and future research can explore other possible mechanisms. We focused on relatedness need satisfaction because leisure nostalgia is primarily social and prior evidence points to relatedness as the key pathway. However, we acknowledge that autonomy and competence—two other core BPNT needs—may also mediate the relationship between leisure nostalgia and life satisfaction. Autonomy could matter when leisure nostalgic memories emphasize agency or choice; competence could matter when they emphasize mastery or achievement. Future studies should test autonomy and competence as mediators.

In addition, emotion may be a relevant mediator. University students may feel positive emotions when they engage in leisure nostalgia, which in turn increase their life satisfaction. In addition, in terms of between-person level, we did not find a mediation process in which leisure nostalgia increased ego depletion, which in turn decreased life satisfaction. However, in terms of within-person level, day-to-day leisure nostalgia may negatively affect individuals' daily life satisfaction through daily ego depletion. Therefore, future researchers could use a diary method to collect information at multiple time points to test the mediating role of ego depletion in the effect of leisure nostalgia on life satisfaction.

Fourth, we tested only one moderator, namely self-regulation capacity. Other individual characteristics may also influence the effects of leisure nostalgia. Previous studies demonstrated that narcissism (Bialobrzeska et al., 2023) played important moderating roles in the effect of nostalgia. Similarly, we argue that the effect of leisure nostalgia may be more positive for people with strong narcissistic traits. Thus, future research could examine individual characteristics that may influence the relationship between leisure nostalgia and life satisfaction. Furthermore, we focused on leisure nostalgia as a general construct and did not distinguish types of nostalgic leisure (e.g., constructive vs. deviant). However, prior research shows different leisure forms can differentially satisfy autonomy, competence, and relatedness—and college students report higher rates of deviant leisure—so this omission limits our ability to know whether the relationship between leisure nostalgia and life satisfaction holds across contexts. Future studies should test leisure-type as a moderator (e.g., whether leisure nostalgia for deviant vs. constructive leisure weakens the relatedness-mediated effect) to clarify the boundary conditions of leisure nostalgia's benefits.

Finally, while our data were collected during the COVID-19 pandemic, we did not obtain measures of contextual variables such as perceived infection risk or isolation intensity, which limits our ability to directly control for their influence. We acknowledge that pandemic-related social restrictions may have amplified experiences of leisure nostalgia. Nevertheless, the key psychological mechanism under investigation—leisure nostalgia satisfying basic psychological needs—is likely to operate beyond this specific context. Leisure nostalgia is not only relevant during crises but also reflects fundamental human needs for continuity and belonging that persist in everyday life. Future research may explore how leisure nostalgia functions as a psychological resource across different social environments, underscoring its long-term significance.

## 5 Conclusions

Based on the basic psychological needs theory and self-regulation theory, we examined the relationship between leisure nostalgia on life satisfaction, as well as the mediating role of relatedness need satisfaction and ego depletion, and the moderating role of self-regulatory capacity in this relationship. We found that relatedness need satisfaction mediated the relationship between leisure nostalgia and university student life satisfaction, and this mediation effect was stronger for students with higher vs. lower self-regulatory capacity. However, the mediating role of ego depletion in the relationship between leisure nostalgia and life satisfaction was not significant. These findings support the basic psychological needs theory and offer a conceptual framework for understanding the mechanisms linking leisure nostalgia to life satisfaction. Moreover, this study provides insights into improving university students' life satisfaction.

## Data Availability

The raw data supporting the conclusions of this article will be made available by the authors, without undue reservation.
